# Influences of Jujube Witches’ Broom (JWB) Phytoplasma Infection and Oxytetracycline Hydrochloride Treatment on the Gene Expression Profiling in Jujube

**DOI:** 10.3390/ijms241210313

**Published:** 2023-06-18

**Authors:** Junqiang Yang, Zhongmei Shen, Pengyan Qu, Rui Yang, Anping Shao, Hao Li, Ailing Zhao, Chunzhen Cheng

**Affiliations:** College of Horticulture/Pomology Institute, Shanxi Agricultural University, Taiyuan 030031, China

**Keywords:** jujube, oxytetracycline hydrochloride, jujube witches’ broom, phytoplasma, gene expression profiling

## Abstract

Jujube witches’ broom disease (JWB), caused by *Candidatus Phytoplasma ziziphi*, is the most destructive phytoplasma disease threatening the jujube industry. Tetracycline derivatives treatments have been validated to be capable of recovering jujube trees from phytoplasma infection. In this study, we reported that oxytetracycline hydrochloride (OTC-HCl) trunk injection treatment could recover more than 86% of mild JWB-diseased trees. In order to explore the underlying molecular mechanism, comparative transcriptomic analysis of healthy control (C group), JWB-diseased (D group) and OTC-HCl treated JWB-diseased (T group) jujube leaves was performed. In total, 755 differentially expressed genes (DEGs), including 488 in ‘C vs. D’, 345 in ‘D vs. T’ and 94 in ‘C vs. T’, were identified. Gene enrichment analysis revealed that these DEGs were mainly involved in DNA and RNA metabolisms, signaling, photosynthesis, plant hormone metabolism and transduction, primary and secondary metabolisms, their transportations, etc. Notably, most of the DEGs identified in ‘C vs. D’ displayed adverse change patterns in ‘D vs. T’, suggesting that the expression of these genes was restored after OTC-HCl treatment. Our study revealed the influences of JWB phytoplasma infection and OTC-HCl treatment on gene expression profiling in jujube and would be helpful for understanding the chemotherapy effects of OTC-HCl on JWB-diseased jujube.

## 1. Introduction

Jujube (*Ziziphus jujuba* Mill.), the most economically important species of Rhamnaceae that has been cultivated in China for more than 7000 years, is one of the oldest cultivated fruit trees in the world [1]. Currently, it is grown in about 50 countries, and its fruit production ranks first among all the dried fruits in China [1,2]. Jujube fruits are rich in nutritional and healthcare values [3,4] and have been frequently used as important raw materials for producing both traditional Chinese and Western medicines and healthcare products [5,6,7]. Jujube has been considered a superfruit for the future mainly owing to its various characteristics (such as nutrient-rich fruit, early and quick harvesting, low cultivation costs, easy management, easy fruit preservation, transportation, etc.) [1,2] and its outstanding tolerance to a wide range of climatic and soil conditions (such as barren soil, salt-alkali, drought, wind and sand conditions, etc.) [1,6,8]. However, the jujube industry has been greatly threatened by the jujube witches’ broom (JWB) disease, the most destructive jujube phytoplasma disease that has destroyed about 3~5% of jujube trees in the world [9].

The pathogen of JWB disease was once thought to be viruses or mycoplasma-like organisms (MLOs) but was finally recognized as phytoplasma named *Candidatus Phytoplasma ziziphi* [10,11]. Typical symptoms caused by JWB in jujube trees included witches’ broom deformity (branch clustering), short internodes in developing tissues, yellowing, abnormally small leaves, phyllody (leaf-like floral organs), dense root suckers and abnormally colored fruits [12,13]. The JWB phytoplasmas are phloem-inhabiting [12]. In jujube petioles with JWB disease, obvious phytoplasmas accumulations were found in sieve elements [14]. Additionally, multivesicular bodies and callose accumulation could be observed in phloem parenchyma cells and sieve elements of JWB-diseased jujube leaves [12]. The phytoplasma infection destroyed grana and stoma lamellae structures of chloroplasts, inhibited photosynthesis, influenced phytohormones metabolism, caused abnormal carbohydrates accumulation, stimulated amino acids biosynthesis, and energy metabolisms of diseased jujube, and finally led to nutrient exhaustion, abnormal growth and even death of the whole tree [14].

The jujube responses to JWB phytoplasma infection were very complex and have been investigated from physiological, biochemical and molecular levels [15]. In addition to suppressed photosynthesis ability and reduced contents of chlorophyll and total soluble sugars, increased activities of antioxidant enzymes and significantly changed biosynthesis of secondary metabolites were also discovered in JWB-diseased jujube trees [15,16]. Of note, the changes in some phytohormones, such as auxin and jasmonate, were reported to be closely related to the symptom development in JWB-infected trees [9,15,17]. The differential expression of many defense genes, such as genes encoding peroxidases, proline-rich proteins, eukaryotes elongation factor1A and some pathogenesis-related (PR) proteins, were reported to be important for the jujube responses to JWB phytoplasma infection [18]. Moreover, flavonoid biosynthesis, phenylalanine metabolism and phenylpropanoid biosynthesis, plant–pathogen interaction, plant hormone signaling transduction, carbon metabolism, photosynthesis, amino acid metabolism and carotenoid biosynthesis-related genes or proteins have been reported to be functional in the jujube–phytoplasmas interactions [15,19].

JWB disease can not only be transmitted by insect vectors but also through grafting, which greatly increases the difficulty of its prevention and control [20]. In the last decade, due to the decreasing price of jujube fruits, insufficient labor and attention were paid to the jujube trees and orchard management, which finally led to the fast spread of JWB disease throughout the jujube production areas in China [21]. Furthermore, due to the fact that the pathogen is unculturable in vitro, research on its pathogenesis progressed quite slowly [19].

For the prevention and control of JWB, a variety of measures have been taken by scientists and farmers, such as resistant rootstock and variety breeding, in addition to physical-, chemical-, biological- and comprehensive controls [17,22]. Among them, antibiotics treatments, especially tetracycline derivatives treatments, have been reported to be quite effective in recovering trees from phytoplasma infection [17,23]. Wang et al. [17] reported that JWB-diseased jujube plantlets exhibited obviously reduced JWB symptoms after 25 mg/L tetracycline treatment for three months, and all JWB symptoms disappeared after six months of treatment. Through PCR analysis using phytoplasma *16S rRNA* specific primers, they found that jujube plantlets became phytoplasma negative at six months post tetracycline treatment, indicating that tetracycline treatment could clear the phytoplasmas in jujube plantlets [17]. Similarly, in one of our previous studies, we found that trunk injection of 10 g/L oxytetracycline into the trunk of mild JBW-diseased trees could achieve a more than 80% curing rate [23]. Recently, oxytetracycline hydrochloride (OTC-HCl), the hydrochloride salt form of oxytetracycline, has been reported to be effective in impairing the accumulation and symptom progression of phytoplasmas in diseased plants [24] and has been successfully applied in controlling phytoplasmas [25] and in producing phytoplasma-free plants [26]. In this study, we investigated the chemotherapy effects of 10 g/L OTC-HCl trunk injection treatment on mild JWB-diseased jujube trees and found that this treatment could recover more than 86% of the diseased trees from phytoplasma infection. In order to explore the underlying molecular mechanism, comparative transcriptomic analysis of healthy control (C group), JWB-diseased (D group) and OTC-HCl treated JWB-diseased (T group) *Z. jujuba* cv. ‘Muzao’ leaves was then performed. Our study will be helpful in understanding the influences of JWB phytoplasma infection and OTC-HCl treatment on gene expression profiling in jujube in the field and can provide a basis for the JWB disease control and disease resistance breeding of jujube in the future.

## 2. Results

### 2.1. Chemotherapy Effects of Oxytetracycline Hydrochloride (OTC-HCl) on Jujube Witches’ Broom Disease (JWB)-Diseased Jujube Trees

**Figure 1:**
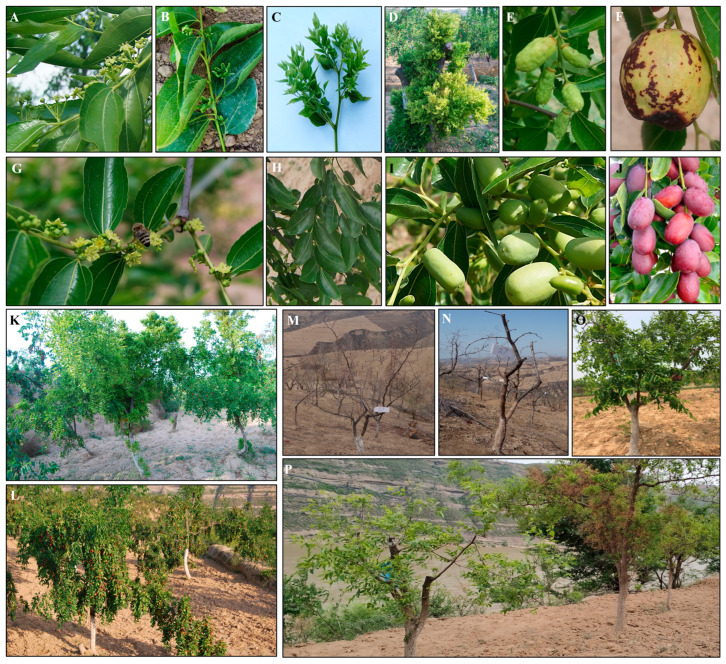
Jujube witches’ broom disease (JWB)-diseased jujube trees before and after oxytetracycline hydrochloride (OTC-HCl) trunk injection treatment: (**A**) prolonged floral shoots of JWB-diseased jujube; (**B**) leaf-like floral organs; (**C**) witches’ broom deformity (branch clustering) symptom; (**D**) dense root suckers and branch clustering; (**E**) abnormal young jujube fruits; (**F**) abnormally colored mature fruit; (**G**) healthy flowers; (**H**) healthy leaves; (**I**) healthy young fruits; (**J**) normal mature fruits; (**K**) JWB-diseased trees with typical witches’ broom deformity symptom; (**L**) healthy jujube trees with high fruit production in the field; (**M**) JWB-diseased jujube tree before OTC-HCl treatment. The witches’ broom branches are not easy to fall off the trunk in winter; (**N**) diseased branches were removed from JWB-diseased jujube trees before OTC-HCl treatment; (**O**) phenotype of OTC-HCl treated JWB-diseased jujube tree at about 20 days post-treatment; (**P**) OTC-HCl treated (left) and untreated (right) JWB-diseased jujube trees in the field at about thirteen months after OTC-HCl trunk injection treatment.

Typical JWB disease symptoms, including prolonged floral shoots (Figure 1A), leaf-like floral organs (Figure 1B), branch clustering (Figure 1C), dense root suckers and branch clustering (Figure 1D), abnormal young fruits (Figure 1E) and abnormally colored mature fruits (Figure 1F), were observed in the field. Compared to the jujube trees with healthy flowers (Figure 1G), leaves (Figure 1H), and young (Figure 1I) and mature fruits (Figure 1J), the fruit production of diseased trees displaying witches’ broom deformity (Figure 1K,L) decreased significantly (Figure 1L). Before leaf sprouting, all the branches showing typical JWB symptoms were removed (Figure 1M,N). OTC-HCl trunk injection was performed after leaf sprouting. At about 20 days post OTC-HCl injection, some treated JWB-diseased trees sprouted out healthy brunches (Figure 1O). Additionally, at thirteen months post OTC-HCl treatment, 52 of the 60 (86.67%) OTC-HCl treated mild JWB-diseased jujube trees recovered to normal morphology (Figure 1P), indicating that OTC-HCl treatment is quite helpful in recovering mild JWB-diseased jujube trees from phytoplasma infection.

### 2.2. RNA-Seq Results and Identification of Differentially Expressed Genes (DEGs)

RNA-seq was performed using leaves from healthy control (C group), JWB-diseased (D group) and OTC-HCl treated JWB-diseased (T group) *Z. jujuba* cv. ‘Muzao’ trees. The results showed that 39,165,042~43,524,986 reads containing 5,828,462,420~6,515,843,862 clean bases were obtained from the nine cDNA libraries (three replications for each group) (Table 1). Their mapping ratio against the *Z. jujuba* genome was all higher than 84%, and more than 73% of them were unique mapped reads. The GC contents of our obtained reads from the nine libraries were all about 45%. Moreover, the Q20 and Q30 values for the nine cDNA libraries were all higher than 96% and 92%, respectively. These results indicated that the quality of our RNA-seq data is high.

**Figure 2:**
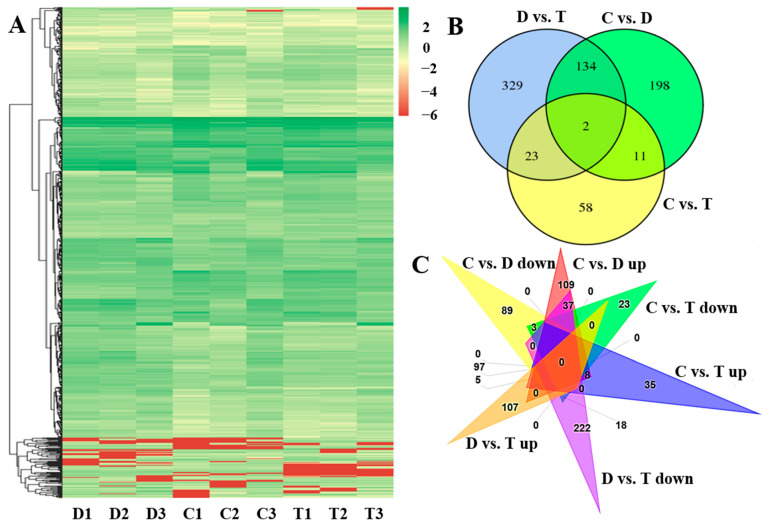
Gene expression heatmap (**A**) for all jujube genes and Venn diagrams (**B**,**C**) for the DEGs identified in this study. For heatmap drawing, the log_2_ (FPKM + 1) values of jujube genes were used. C1–C3, D1–D3 and T1–T3 were three biological replications for C, D and T groups, respectively. The ‘up’ and ‘down’ in the Venn diagram C represent up-regulated and down-regulated genes in corresponding comparisons, respectively.

A total of 32,084 genes, including 29,293 known genes and 2791 new genes, were identified to express in at least one cDNA library. Among them, 755 (including 688 known genes and 67 new genes) were identified as DEGs in at least one of the three comparisons (‘C vs. D’, ‘C vs. T’ and ‘D vs. T’) under the criteria of |log_2_ (fold change)| ≥ 1 and *p*-value < 0.05. There were 345 genes (156 up-regulated and 189 down-regulated), 94 genes (50 up-regulated and 44 down-regulated) and 488 (210 up-regulated and 278 down-regulated) genes identified as DEGs in ‘C vs. D’, ‘C vs. T’ and ‘D vs. T’ (Figure 2A), respectively. The lowest account of DEGs was identified in ‘C vs. T’, indicating that the transcriptome profile of OTC-HCl treated JWB-diseased jujube (the T group) was similar to that of the healthy control (the C group). Among the 755 DEGs, 198 (109 up-regulated and 89 down-regulated), 329 (107 up-regulated and 222 down-regulated) and 58 (35 up-regulated and 23 down-regulated) DEGs were ‘C vs. D’-, ‘C vs. T’- and ‘D vs. T’-specific (Figure 2B), respectively. There were 134, 23 and 11 common DEGs for ‘C vs. D’ and ‘D vs. T’, ‘C vs. T’ and ‘D vs. T’, and ‘C vs. D’ and ‘C vs. T’ (Figure 2B), respectively. Interestingly, all the 134 common DEGs identified between ‘C vs. D’ and ‘D vs. T’ showed the opposite change pattern (37 up-regulated in ‘C vs. D’ but down-regulated in ‘D vs. T’, and 97 down-regulated in ‘C vs. D’ but up-regulated in ‘D vs. T’ ), but all the 23 common DEGs (5 up-regulated and 18 down-regulated) identified between ‘C vs. T’ and ‘D vs. T’ and all the 11 common DEGs (3 up-regulated and 8 down-regulated) identified between ‘C vs. D’ and ‘C vs. T’ showed the same change patterns (Figure 2C). Moreover, two genes (*purple acid phosphatase 17* (*PAP17*, gene11700, up-regulated in ‘C vs. D’ and ‘C vs. T’ but down-regulated in ‘D vs. T’) and *NDH-dependent cyclic electron flow 1* (*NDF1*, gene30294, up-regulated in all the three comparisons)) were identified as common DEGs of all the three comparisons (Figure 2B).

### 2.3. Gene Ontology (GO) Enrichment Analysis Results of DEGs

GO enrichment analysis showed that, by using *p*-value < 0.05 as a criterium, there were 41 biological process (BP) terms, 16 cellular component (CC) terms and 31 molecular function (MF) terms significantly enriched in ‘C vs. D’; 34 BP terms, 6 CC terms and 25 MF terms significantly enriched in ‘C vs. T’; and 59 BP terms, 22 CC terms and 43 MF terms significantly enriched in ‘D vs. T’ (Supplemental Table S1). Fifteen BP terms (including ‘nucleosome assembly’, ‘dephosphorylation’, ‘chromatin silencing’, ‘response to biotic stimulus’, ‘cellulose catabolic process’, etc.) were significantly enriched in both ‘C vs. D’ and ‘D vs. T’; 12 BP terms (including ‘cellular protein catabolic process’, ‘phosphate ion homeostasis’, ‘regulation of phosphate transmembrane transport’, ‘sulfate transport’, etc.) were significantly enriched in both ‘C vs. T’ and ‘D vs. T’; and only one BP term (‘tetrahydrofolate metabolic process’) was significantly enriched in both ‘C vs. D’ and ‘C vs. T’. Notably, DEGs involved in ‘cellular response to phosphate starvation’ were significantly enriched in all three comparisons.

Among the significantly enriched CC terms, six terms (including ‘nucleosome’, ‘extracellular region’, ‘plastid’, ‘nuclear chromatin’, ‘heterochromatin’ and ‘chloroplast thylakoid membrane’) were found to be significantly enriched in both ‘C vs. D’ and ‘D vs. T’ comparisons, and no other GO term was significantly enriched in two or three comparisons.

Among the significantly enriched MF terms, 19 terms (such as ‘protein heterodimerization activity’, ‘nucleosomal DNA binding’, ‘acid phosphatase activity’, ‘chromatin binding’, ‘cellulase activity’, ‘iron ion binding’, ‘intramolecular transferase activity’, etc.) were significantly enriched in ‘C vs. D’ and ‘D vs. T’, and only one MF term (‘sarcosine oxidase activity’) was significantly enriched in ‘C vs. D’ and ‘C vs. T’.

### 2.4. Kyoto Encyclopedia of Genes and Genomes (KEGG) Enrichment Analysis Results of DEGs

KEGG enrichment analysis of DEGs was also performed (Supplemental Table S2). Results showed that DEGs from ‘C vs. D’ were significantly enriched in ‘Sesquiterpenoid and triterpenoid biosynthesis’, ‘Pentose phosphate pathway’, ‘Glycosphingolipid biosynthesis-globo series’, ‘Taurine and hypotaurine metabolism’ and ‘Limonene and pinene degradation’ pathways; DEGs from ‘C vs. T’ were significantly enriched in ‘Protein processing in endoplasmic reticulum’, ‘Endocytosis’, ‘Inositol phosphate metabolism’ and ‘Phosphatidylinositol signaling system’ pathways; and DEGs from ‘D vs. T’ were significantly enriched in ‘Photosynthesis’, ‘Taurine and hypotaurine metabolism’, ‘Arachidonic acid metabolism’, ‘Glutathione metabolism’, ‘Ubiquinone and other terpenoid-quinone biosynthesis’, ‘Sesquiterpenoid and triterpenoid biosynthesis’, and ‘Cyanoamino acid metabolism’ pathways. Notably, the ‘Taurine and hypotaurine metabolism’ and ‘Sesquiterpenoid and triterpenoid biosynthesis’ pathways were found to be significantly enriched by DEGs from both ‘C vs. D’ and ‘D vs. T’.

### 2.5. MapMan Annotation Results of DEGs

By using MapMan, all the 755 DEGs were further annotated. The results showed that they could be assigned to 29 main Bin pathways, with the ‘not assigned’ pathway consisting of the largest number of DEGs (in a total of 196) (Table 2 and Supplemental Table S3). Seven of the DEGs encoding Major allergen Mal d1 proteins were found to be down-regulated in ‘C vs. D’ but significantly up-regulated in ‘D vs. T’; three (gene3362, gene8763 and gene8766) of the four DEGs encoding 23 kDa jasmonate-induced proteins (JIPs) were down-regulated in ‘C vs. D’ but up-regulated in ‘D vs. T’; and three DEGs encoding Mediator complex subunit Med2 proteins were found to be JWB inducible. Moreover, three *D-arabinono-1,4-lactone oxidase family genes* (gene27437, gene27438 and gene27914), two *BURP domain-containing protein* genes (gene18500 and gene18503) and two *polyphenol oxidase* (*PPO*) genes (gene27589 and gene27870) were down-regulated in ‘C vs. D’ but up-regulated in ‘D vs. T’.

A total of 88 DEGs were assigned to the ‘misc’ pathway (Supplemental Table S3), suggesting that they might have miscellaneous functions. Three DEGs (gene11489, gene22048 and gene22051) encoding glutathione S transferases (GSTs) and many DEGs encoding UDP glucosyl and glucoronyl transferases, acid and other phosphatases, O-methyl transferases, alpha/beta-Hydrolases superfamily proteins, Aldolase-type TIM barrel family protein, and beta-1,3-glucanase 2 were up-regulated in ‘C vs. D’ but down-regulated in ‘D vs. T’. Moreover, nine DEGs encoding gluco-, galacto- and mannosidases were down-regulated in ‘C vs. D’ but up-regulated in ‘D vs. T’.

**Figure 3:**
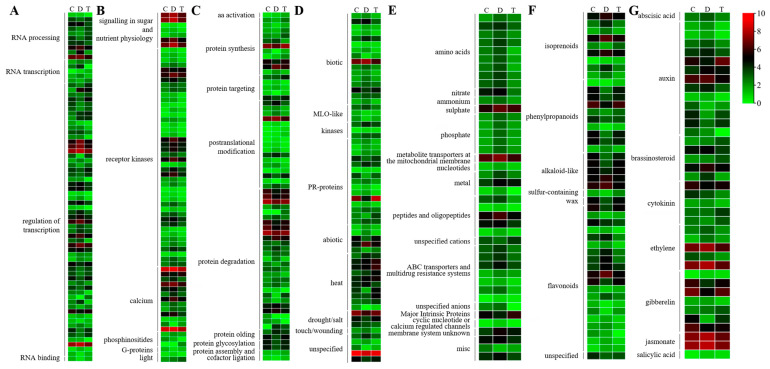
Gene expression heatmaps for the DEGs involved in ‘RNA’ (**A**), ‘signalling’ (**B**), ‘protein’ (**C**), ‘stress’ (**D**), ‘transport’ (**E**), ‘secondary metabolism’ (**F**) and ‘hormone metabolism’ (**G**) pathways. For the heatmap drawing, the genes’ average FPKM (Fragments Per Kilobase of exon model per Million mapped fragments) values of three biological replications from the same group were used. The redder the color, the higher the expression level; the greener, the lower the expression level.

Seven pathways, including ‘RNA’, ‘signalling’, ‘protein’, ‘stress’, ‘transport’, ‘secondary metabolism’ and ‘hormone metabolism’, were assigned with more than thirty DEGs (Figure 3 and Supplemental Table S3). Among the 72 ‘RNA’-related DEGs (Figure 3A), almost all the DEGs encoding WRKY transcription factors were up-regulated by phytoplasma infection but down-regulated after OTC-HCl injection treatment. Sixty-one of the 65 ‘signalling’-related DEGs were up-regulated in ‘C vs. D’, and most of them were down-regulated in ‘D vs. T’ (Figure 3B). Of the 62 ‘Protein’-related DEGs, most of the protein synthesis-, protein glycosylation and protein assembly-, and cofactor ligation-related DEGs were down-regulated after OTC-HCl injection treatment, while most of the DEGs involved in protein posttranslational modification were up-regulated by phytoplasma infection but down-regulated after OTC-HCl injection treatment (Figure 3C). Of the 62 ‘Stress’-related DEGs, most of the biotic stress-related DEGs expressed the highest in D group (Figure 3D). Additionally, 11 of the 15 DEGs encoding PR proteins were up-regulated in ‘C vs. D’. However, two (gene16269 and gene30267) of the three DEGs encoding homologs of the barley mildew resistance locus o (MLO) proteins showed the highest expression in C, followed by in T. Forty-six of the 49 ‘Transport’-related DEGs showed the highest expression in C (Figure 3E). All the DEGs encoding proteins functioning in the transportation of amino acids, nitrate, ammonium, sulfate, phosphate, nucleotides, metal, peptides and oligopeptides, and ABC transporters and multidrug resistance systems were up-regulated in ‘C vs. D’ (Figure 3E). Among the 37 ‘Secondary metabolism’-related DEGs, most of the isoprenoid biosynthesis-related genes were up-regulated in ‘C vs. D’ but down-regulated in ‘D vs. T’ (Figure 3F). However, almost all the phenylpropanoid biosynthesis-related DEGs showed the opposite change patterns (Figure 3F). Additionally, the ‘Hormone metabolism’-related DEGs consisted of 13, 7, 5, 5, 4, 2, 1 and 1 genes involved in the metabolism of auxin, gibberellin, cytokinin, ethylene, brassinosteroid, jasmonate, abscisic acid and salicylic acid (Figure 3G), respectively.

**Figure 4:**
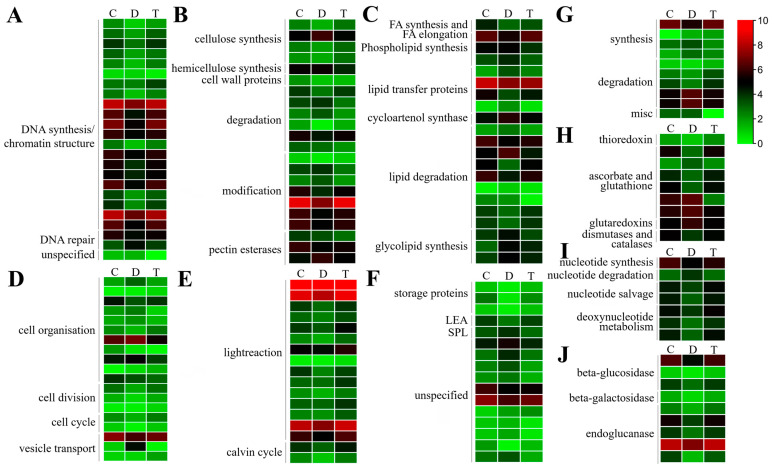
Gene expression heatmaps for the DEGs involved in ‘DNA’ (**A**); ‘cell wall’ (**B**); ‘lipid metabolism’ (**C**); ‘cell’ (**D**); ‘PS’ (**E**); ‘development’ (**F**); ‘amino acid metabolism’ (**G**); ‘redox’ (**H**); ‘nucleotide metabolism’; (**I**) and ‘misc.gluco-, galacto- and mannosidases’ (**J**) pathways. PS: photosynthesis. For the heatmap drawing, the genes’ average FPKM values of three biological replications from the same group were used. The redder the color, the higher the expression level; the greener, the lower the expression level.

There were 22, 20, 20, 19, 17, 16, 9 and 9 DEGs assigned to ‘DNA’, ‘cell wall’, ‘lipid metabolism’, ‘cell’, ‘PS (photosynthesis)’, ‘development’, ‘amino acid metabolism’ and ‘redox’ (Figure 4, Supplemental Table S3), respectively. Most of the ‘DNA’ and ‘cell wall’ related DEGs expressed the lowest in JWB-diseased jujube leaves (Figure 4A,B). Of the 20 ‘lipid metabolism’-related DEGs, 11 were up-regulated, and 9 were down-regulated in JWB-diseased jujube (Figure 4C). Most of the cell organization-related DEGs were up-regulated, while all the cell division and cell cycle-related DEGs were up-regulated in JWB-diseased jujube (Figure 4D). All the ‘PS’-related DEGs were up-regulated after OTC-HCl injection treatment (Figure 4E). Regarding the ‘development’-related DEGs, all the DEGs encoding storage proteins and LEAs (late embryogenesis abundant proteins) expressed the lowest in JWB-diseased jujube, and their expression was up-regulated after OTC-HCl injection treatment (Figure 4F). Moreover, most of the other ‘development’-related DEGs were up-regulated by phytoplasma infection (Figure 4F). Three of the four amino acid biosynthesis-related DEGs were up-regulated, while three amino acid degradation-related DEGs were down-regulated in JWB-diseased jujube (Figure 4G). Furthermore, there were four and five ‘Redox’ related DEGs that were up-regulated and down-regulated by JWB phytoplasma infection (Figure 4H), respectively, and their expression levels in T were similar to that in C.

All the other twelve BIN pathways consisted of less than eight DEGs (Table 2, Supplemental Table S3). It is interesting that, of the seven ‘nucleotide metabolism’-related DEGs (Figure 4I), six were greatly down-regulated in D but up-regulated after OTC-HCl treatment.

### 2.6. PageMan Enrichment Analysis Results of DEGs

**Figure 5:**
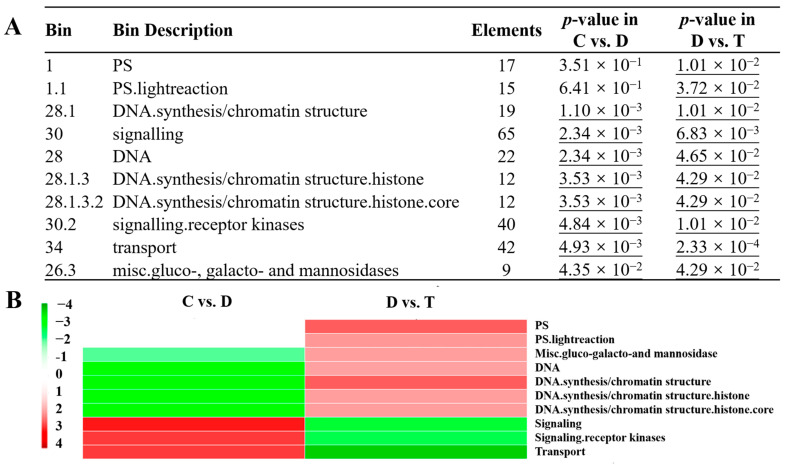
PageMan enrichment analysis results of DEGs identified in this study: (**A**) Pathways significantly enriched by DEGs from ‘C vs. D’ and ‘D vs. T’. No significantly enriched pathway was identified in ‘C vs. T’. The underlined values represent significant enrichment (*p*-value < 0.05); (**B**) Bin-wise Wilcoxon test analysis results of DEGs. For the Bin-wise Wilcoxon test, Benjamini and Hochberg adjusted *p*-value < 0.05 was used as criterium for screening significantly enriched pathways. The redder the color, the much more significant up-regulation; the greener, the much more significant down-regulation.

By using the PageMan software embedded in MapMan, pathway enrichment analysis of all DEGs was performed (Table 2). The results showed that these DEGs were significantly enriched in seven pathways (‘DNA.synthesis/chromatin structure’; ‘signalling’; ‘DNA’; ‘DNA.synthesis/chromatin structure.histone’; ‘DNA.synthesis/chromatin structure.histone.core’; ‘signalling.receptor kinases’; ‘transport’; and ‘misc.gluco-, galacto- and mannosidases’ (Figure 4J)) in ‘C vs. D’ (Figure 5A). No significant enriched pathway was identified in ‘C vs. T’. In ‘D vs. T’, besides the seven significantly enriched pathways in ‘C vs. D’, ‘PS’ and ‘PS.lightreaction’ pathways were also significantly enriched, and DEGs involved in these two pathways were all up-regulated in T compared to D. This indicated that the expression of photosynthesis-related genes in leaves of JWB-diseased jujube trees was restored after OTC-HCl injection treatment.

**Figure 6:**
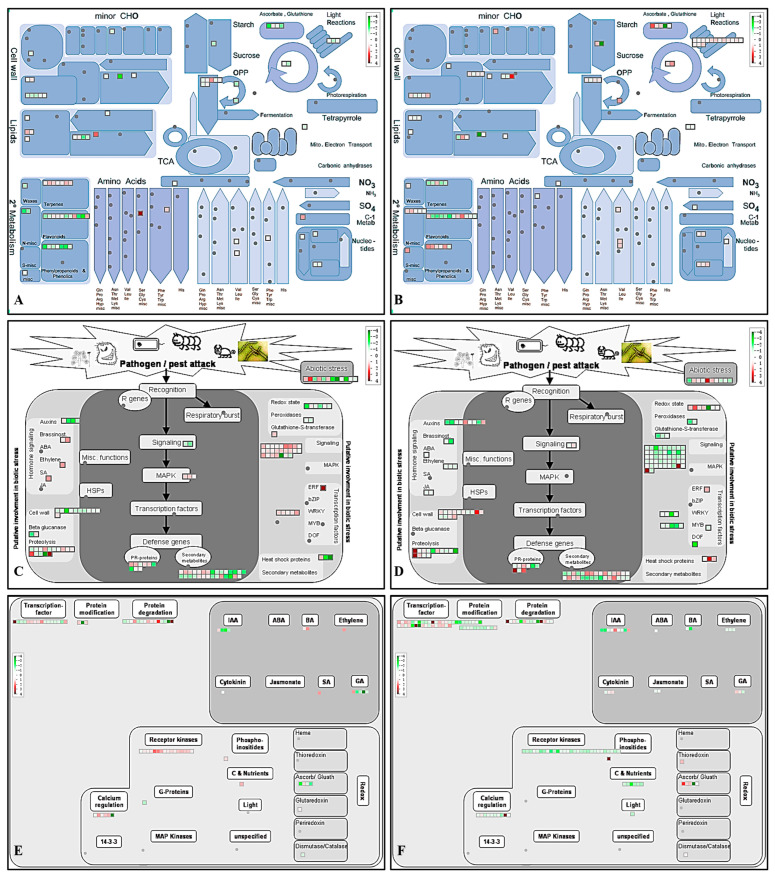
Overview figures for metabolism-, stress- and regulation-related DEGs identified in ‘C vs. D’ and ‘D vs. T’ comparisons: (**A**,**B**) metabolism overview figure for DEGs identified in ‘C vs. D’ and ‘D vs. T’, respectively. (**C**,**D**) stress overview figure for for DEGs identified in ‘C vs. D’ and ‘D vs. T’, respectively. (**E**,**F**) regulation overview figure for DEGs identified in ‘C vs. D’ and ‘D vs. T’, respectively. The DEGs showed overwhelmingly opposite change patterns in ‘C vs. D’ and ‘D vs. T’. All these figures were generated using MapMan. Red boxes represent up-regulated genes, and green boxes represent down-regulated genes. The redder, the greater fold change the gene was up-regulated. The greener, the greater fold change the gene was down-regulated.

In order to show the differential expression patterns of DEGs identified in ‘C vs. D’ and ‘D vs. T’ better, a Bin-wise Wilcoxon test was performed using Benjamini and Hochberg adjusted *p*-value < 0.05 as criterium (Figure 5B). The results showed that DEGs involved in ‘PS’ and ‘PS.lightreaction’ were mainly up-regulated in ‘D vs. T’. Moreover, DEGs involved in ‘DNA.synthesis/chromatin structure’; ‘DNA’; ‘DNA.synthesis/chromatin structure.histone’; ‘DNA.synthesis/chromatin structure.histone.core’; and ‘misc.gluco-, galacto- and mannosidases’ were down-regulated in ‘C vs. D’ but overwhelmingly up-regulated in ‘D vs. T’. Additionally, the DEGs involved in ‘signalling’, ‘signalling.receptor kinases’ and ‘transport’ pathways also exhibited adverse change patterns between the two comparisons, i.e., mostly up-regulated in ‘C vs. D’ but down-regulated in ‘D vs. T’. Consistently, the metabolism (Figure 6A,B), stress (Figure 6C,D), and regulation (Figure 6E,F) overview figures of DEGs identified in the two comparisons can also reflect the opposite changes in DEGs involved in photosynthesis-, signaling- and some other pathways.

### 2.7. Quantitative Real-Time PCR (qRT-PCR) Analysis Results

**Figure 7:**
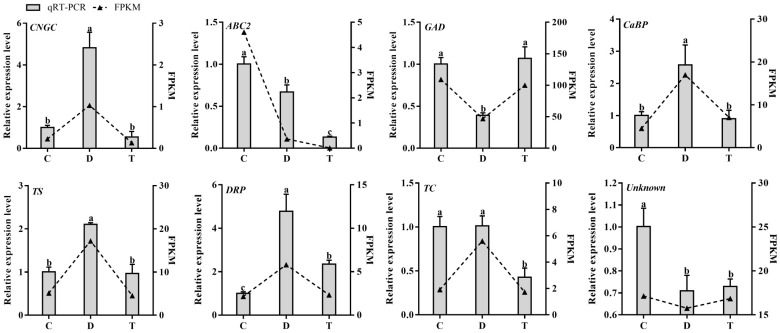
Gene expression analysis results of eight selected genes in leaves of healthy (C), JWB-diseased (D) and OTC-HCl treated JWB-diseased jujube trees (T) using transcriptome data and qRT-PCR. CNGC: cyclic nucleotide-gated channel; ABC2: ATP-binding cassette transporter 2; GAD: glutamate decarboxylase; CaBP: calcium-binding EF-hand family protein; TS: terpene synthase; DRP: CC-NBS-LRR class disease resistance protein; TC: terpenoid cyclase; Unknown: unknown protein. Different letters (a, b and c) above columns represent significant differences among samples at *p* < 0.05 level.

Quantitative real-time PCR analysis was performed to validate the expression of eight selected genes (Figure 7). According to the transcriptome data, *Cyclic nucleotide-gated channel* (*CNGC*, gene7282), *Calcium-binding EF-hand family protein* (*CaBP*, gene13237), *Terpene synthase* (*TS*, gene14606), *CC-NBS-LRR class disease resistance protein* (*DRP*, gene25582) and *Terpenoid cyclase* (*TC*, gene14697) genes all expressed the highest in D. Our qRT-PCR results also revealed that the expression levels of *CNGC* (gene7282), *CaBP* (gene13237), *TS* (gene14606) and *DRP* (gene25582) in leaves of D group were all significantly higher than that in leaves of C and T groups. Notably, the expression levels of *CNGC* and *DRP* in D were both more than four times higher than that in C. Although the expression levels of *TC* (gene14697) in D are similar to that in C, its expression in D was also significantly higher than in T. Both the transcriptome and qRT-PCR results revealed that the expression levels of *ATP-binding cassette transporter 2* (*ABC2*, gene9336) followed the order: C > D > T; the expression of *Glutamate decarboxylase* (*GAD*, gene11898) was the lowest in D; and the *unknown protein* (gene19644) expressed the highest in C, and its expression levels in D and T were similar. These results showed that our qRT-PCR results were mostly consistent with the transcriptome data, indicating that our transcriptome data were believable.

## 3. Discussion

The jujube industry is greatly threatened by the JWB disease. In order to cope with this disease, many methods were applied [17,22], and the tetracycline derivatives treatments were confirmed to be quite effective [17,23]. In this study, we reported the chemotherapy effects of OTC-HCl in recovering more than 86% of the mild JWB-diseased trees in the field. To explore the underlying molecular mechanism, comparative transcriptomic analysis using leaves from JWB-diseased, OTC-HCl treated JWB-diseased and healthy control jujube trees was performed. In total, 755 DEGs (including 488 in ‘C vs. D’, 345 in ‘D vs. T’, and 94 in ‘C vs. T’) were identified among the three groups. Based on the results obtained in this study, the following was revealed.

### 3.1. Phytoplasma Infection Influenced Greatly the DNA and RNA Metabolisms and Signaling in Jujube Leaves

Our GO enrichment analysis results revealed that DEGs involved in ‘nucleosome assembly’, ‘dephosphorylation’ and ‘chromatin silencing’ were significantly enriched in both ‘C vs. D’ and ‘D vs. T’, suggesting that the gene expression profiling in jujube leaves were greatly influenced by phytoplasma infection. Moreover, by using MapMan, 22 and 72 DEGs were annotated to be ‘DNA’- and ‘RNA’-related, respectively. Eighteen of the 22 ‘DNA’ related DEGs expressed the lowest in JWB-diseased jujube, but almost all the ‘RNA’ related DEGs, such as genes encoding WRKY transcription factors, were up-regulated by phytoplasma infection but down-regulated after OTC-HCl injection treatment. These results indicated that the DNA metabolism of jujube was inhibited, but some RNA transcription and regulation reactions were induced by phytoplasma infection.

Receptor kinases were major signaling receptors and were mostly up-regulated by disease infection [27]. Consistently, in our study, DEGs involved in ‘signalling.receptor kinases’ were mostly up-regulated in JWB-diseased trees but down-regulated after OTC-HCl treatment. Calcium-binding proteins were demonstrated to play important roles in plant responses to both abiotic and biotic stresses [28,29,30]. In our study, many *CaBPs* were found to be induced by phytoplasma infection and down-regulated after OTC-HCl treatment. Our qRT-PCR analysis also verified the significant induction of a *CaBP* gene (gene13237) by phytoplasma infection, suggesting that it might function in the jujube–phytoplasma interactions.

Calcium signaling-related genes were related to reactive oxygen species (ROS) signaling [31,32]. The ROS accumulation in phytoplasma-infected jujube leaves was reported to be much higher [33]. In this study, nine ‘Redox’-related DEGs were identified, and their expression in JWB-diseased jujube leaves recovered after OTC-HCl injection treatment, suggesting that the ROS signaling was involved in the phytoplasma attack response and the high ROS accumulation damages caused by the phytoplasma infection were alleviated after OTC-HCl treatment. Furthermore, we also identified 49 ‘stress’-related DEGs (including 15 *PR* genes) that are mostly up-regulated in JWB-diseased trees. However, two *MLO* genes were found to be down-regulated in diseased trees. MLOs have been demonstrated to be negative regulators of plant immunity [34] and sensitivity to ROS-induced gene expression [29]. Their down-regulation in JWB-diseased trees might be helpful for the scavenging of highly accumulated ROS.

CNGCs play important roles in signal transduction during plant stress responses [35]. According to our transcriptome data, the expression of a *CNGC* (gene7282) was found to be significantly higher in JWB-diseased jujube leaves than that in both healthy control and OTC-HCl treated JWB-diseased jujube leaves. Additionally, our qRT-PCR results revealed that its expression in JWB-diseased jujube leaves was more than four-fold of the healthy controls, indicating that its high expression might be important for the jujube signal transduction in response to phytoplasma infection.

### 3.2. OTC-HCl Treatment Alleviated the Influences of Phytoplasma Infection on the Expression of Both Primary and Secondary Metabolites Metabolisms and Their Transportation-Related Genes

JWB infection can greatly inhibit photosynthesis, enhance carbohydrate accumulation and influence the energy metabolism in jujube leaves [14]. Consistently, in our study, the expression levels of all the ‘PS’ related DEGs and all the nine DEGs encoding gluco-, galacto- and mannosidases were suppressed by the phytoplasma infection. We also identified three DEGs involved in major CHO metabolism, three in glycolysis, three in mitochondrial electron transport/ATP synthesis, and two in OPP pathways. Moreover, DEGs involved in signaling in sugar and nutrient physiology and light signaling were all up-regulated in JWB-diseased leaves. Therefore, it was deduced that the photosynthesis ability and the carbohydrate metabolism of JWB-diseased trees recovered after OTC-HCl treatment.

DEGs involved in metabolites metabolisms, such as ‘Sesquiterpenoid and triterpenoid biosynthesis’, ‘Pentose phosphate pathway’, ‘Taurine and hypotaurine metabolism’ and ‘Limonene and pinene degradation’, were significantly enriched by DEGs identified in JWB-diseased trees. Moreover, DEGs involved in ‘Photosynthesis’, ‘Taurine and hypotaurine metabolism’, ‘Arachidonic acid metabolism’, ‘Glutathione metabolism’, ‘Ubiquinone and other terpenoid-quinone biosynthesis’, ‘Sesquiterpenoid and triterpenoid biosynthesis’, and ‘Cyanoamino acid metabolism’ were significantly enriched in ‘D vs. T’. It was noted that most of the protein biosynthesis, protein glycosylation and protein assembly, and cofactor ligation-related DEGs in JWB-diseased trees were up-regulated after OTC-HCl injection treatment, while most of the DEGs involved in protein degradation and posttranslational modification showed opposite change patterns. These results indicated that both the metabolisms of primary and secondary metabolites were greatly affected by phytoplasma infection, and OTC-HCl treatment could recover the protein metabolism changes in JWB-diseased trees.

It was discovered that phytoplasma infection would stimulate amino acids, carbohydrate and energy metabolisms in diseased jujube leaves and finally lead to the exhaustion of nutrients [14]. The differential expression of amino acid metabolism and the carotenoid pathway-related genes was reported to be closely related to the appearance of nutrition deficiency-like symptoms in JWB-diseased jujube trees [19]. In our present study, 49 ‘transport’-related DEGs were found to be overwhelmingly up-regulated in JWB-diseased trees but down-regulated after OTC-HCl treatment. Notably, all the DEGs encoding proteins functioning in the transportation of amino acids, nitrate, ammonium, sulfate, phosphate, nucleotides, metal, peptides and oligopeptides, and ABC transporters and multidrug resistance systems were up-regulated in JWB-diseased jujube compared to healthy control. Therefore, it was deduced that the nutrition deficiency-like symptoms caused by phytoplasma infection might be related to the transportation disorders of these metabolites. Recently, the amino acid has been recognized to be closely related to the stress signaling and defense responses of organisms [36]. In our study, three amino acid biosynthesis-related genes were up-regulated, while three amino acid degradation-related DEGs were down-regulated in JWB-diseased jujube leaves. Our qRT-PCR results also verified the down-regulation of an amino acid metabolism-related *GAD* (gene11898) in JWB-diseased jujube leaves. We also found that DEGs involved in ‘cellular response to phosphate starvation’ were significantly enriched in all three comparisons, suggesting that the nutrient absorption was greatly inhibited by JWB phytoplasma infection.

Interestingly, almost all the isoprenoid biosynthesis-related DEGs were up-regulated in JWB-diseased jujube. However, almost all the phenylpropanoid biosynthesis-related DEGs showed adverse change patterns. These suggested that the secondary metabolite accumulations were also greatly affected by JWB disease. Both our transcriptome and qRT-PCR results showed that the expression of two isoprenoid biosynthesis-related genes, *TC* (gene14697) and *TS* (gene14606), expressed the highest in JWB-diseased jujube leaves. Terpene synthase and terpenoid cyclase play vital roles in the terpenoid metabolism and disease resistance responses of plants [37,38]. Therefore, it can be predicted that the two genes might function in the jujube–JWB phytoplasmas interactions by regulating terpenoid biosynthesis.

### 3.3. Phytohormones Play Important Roles in the Jujube–Phytoplasmas Interactions

Phytohormones are closely involved in plant responses to phytoplasma infection [39]. In JWB phytoplasma infected jujube, decreased auxin and SA accumulations [9] and increased JA [15,17] and zeatin [9] accumulations have been reported. Moreover, the zeatin-to-auxin ratio changes [9] were also demonstrated to play important roles in the jujube–phytoplasmas interactions. In this study, we identified 38 DEGs involved in ‘Hormone metabolism’ in JWB-diseased jujube leaves. It was noted that their expression levels in OTC-HCl-treated JWB-diseased jujube leaves were similar to the healthy ones, suggesting that OTC-HCl restored the phytohormones imbalance in JWB-diseased jujube [17]. *JIP* genes have been reported to be down-regulated at the transcriptional level during JWB recovery [17]. In this study, four differentially expressed *JIPs* were identified in JWB-diseased and OTC-HCl treated JWB-diseased jujube trees, indicating that these *JIPs* play important roles in the jujube response to phytoplasma infection and their differential expression might be resulted from the JA accumulation changes in jujube leaves caused by phytoplasma infection [17].

In this study, many genes encoded unknown proteins, and some new genes were also identified as DEGs. Additionally, we also identified many DEGs that were classified into functional not assigned (such as *NDF1*, *Major allergen Mal d1*, *Mediator complex subunit Med2*, *PPOs*, *D-arabinono-1,4-lactone oxidase family proteins*, *BURP domain-containing proteins*, etc.) and proteins with miscellaneous functions (such as *PAP17*, *GSTs*, etc.). Their functions in the jujube–JWB phytoplasmas interactions can be further studied in the future.

## 4. Materials and Methods

### 4.1. Plant Materials and Antibiotics Used in this Study

The 20-year-old jujube trees (*Z. jujuba* cv. ‘Muzao’) used in this study are grown in Hehuili Village (110°25′58.75″ E; 36°44′6.56″ N), Wanghaisi Town, Yonghe County, Linfen City, Shanxi Province, China. Jujube trees displaying obvious JWB typical symptoms were subjected to DAPI staining and fluorescence microscopy analysis to confirm phytoplasma infection in new shoots. The JWB infection ratio of *Z. jujuba* cv. ‘Muzao’ trees in the jujube orchards we surveyed ranged from 5% to 87.5%, and less than 10% of the normal-looking jujube trees were detected to be JWB positive. After JWB phytoplasma infection detection, a jujube orchard with no severely diseased trees and a JWB infection ratio of about 5% was selected for OTC-HCl treatments. Mild JWB-diseased jujube trees with stem diameters ranging from 8 to 12 cm were subjected to OTC-HCl trunk injection treatment at two to four weeks before leaf sprouting. The chemically synthesized OTC-HCl powders used in this study were purchased from the Feed and Veterinary Medicine Science and Technology Service Department of Yonghe County, Shanxi Province of China. Powders were dissolved in purified water to a final concentration of 10 g/L before use [23].

### 4.2. OTC-HCl Treatments

Before trunk injection, all the branches displaying typical JWB symptoms were removed. At the leaf-spreading period, two injection holes with diameters of about 0.5 cm were drilled 2~3 cm into the xylem of each trunk at 20~30 cm above the ground (with an angle of about 45° to facilitate injection). The two injection holes were drilled on opposite sides with a height interval of about 5~10 cm. Injection bags containing freshly prepared 10 g/L OTC-HCl solution were placed about 1.2 m above the injection holes after leaf emergence. For each tree, 1.5 L OTC-HCl solutions were injected into the trunk in 24 h. Totally, 60 mild JWB-diseased trees were treated with this method. A schematic illustration of the treatment is shown in Supplemental Figure S1. Normally winter pruned healthy jujube trees grown in the same orchard was used as controls.

Thirteen months post-OTC-HCl treatment, the phenotypes of branches, leaves, flowers and fruits were observed and photographed to evaluate the chemotherapy effects of OTC-HCl treatment. The curing rate of OTC-HCl treatment was calculated using the formula of curing rate (%) = number of recovered trees/number of treated JWB − diseased trees × 100%.

### 4.3. RNA Isolation and Transcriptome Assembly

Fully expanded leaves of JWB-diseased (D group), OTC-HCl treated JWB-diseased (T group) and healthy control (C group) *Z. jujuba* cv. ‘Muzao’ trees were harvested at the same time, taken back to the laboratory on ice, washed three times using sterile water, and stored in a −80 °C freezer for later use. Total RNA was extracted from leaves using RNA simple Total RNA Kit (Tiangen, Beijing, China). In order to determine the integrity and purity of isolated RNA, 1% agarose gel electrophoresis detection and nanodrop spectrophotometry method was applied, respectively. High-quality RNA samples were sent to Beijing Biomarker Biotechnology Company (Beijing, China) for RNA-seq on the Illumina HiSeq™2000 platform (San Diego, California, USA). For each group, three biological replications were made.

### 4.4. Identification of Differentially Expressed Genes (DEGs)

After removing low-quality reads from raw data, the remaining clean reads were mapped to the jujube genome (ftp://ftp.ncbi.nlm.nih.gov/genomes/Ziziphus_jujuba/, accessed on 12 December 2022) using hisat2 software version 2.0.4 [40]. For gene expression analysis, the transcript abundances of genes were firstly normalized into FPKM (Fragment per kilobase of exon model million mapped reads) [41]. Then, differentially expressed genes (DEGs) among C, D and T groups were screened using edgeR [42] with criteria of |log_2_ (fold change)| ≥ 1 and *p*-value < 0.05.

### 4.5. Enrichment Analysis of DEGs

GO and KEGG enrichment analysis of DEGs were performed using GOseq R packages based on Wallenius non-central hyper-geometric distribution and KOBAS software, respectively. GO terms and pathways with corrected *p*-values < 0.05 were considered as significantly enriched. All the *Z. jujuba* CDS sequences were submitted to Mercator v.3.6 (https://plabipd.de/portal/mercator-sequence-annotation, accessed on 7 January 2023) to obtain the mapping file used for MapMan analysis [43]. Pathway enrichment analysis was performed by using PageMan embedded in MapMan with *p*-value < 0.05 as a criterium.

### 4.6. Quantitative Real-Time PCR (qRT-PCR) Analysis

The same total RNA samples used for RNA-seq were used for gene expression validation of eight selected genes (including five phytoplasma infection-induced DEGs (*Cyclic nucleotide-gated channel* (*CNGC*, gene7282), *Calcium-binding EF-hand family protein* (*CaBP*, gene13237), *Terpenoid cyclase* (*TC*, gene14697), *Terpene synthase* (*TS*, gene14606), *CC-NBS-LRR class disease resistance protein* (*DRP*, gene25582)), one DEG (*ATP-binding cassette transporter 2* (*ABC2*, gene9336)) that down-regulated in both D and T, one phytoplasma infection down-regulated DEG (*Glutamate decarboxylase* (*GAD*, gene11898)), and one *unknown protein* gene (gene19644) that did not show significant change among the three groups) using quantitative real-time PCR (qRT-PCR). First-strand cDNA was transcribed from 1 μg leaf total RNA using the TUREscript 1st Stand cDNA Synthesis Kit (Adlab, Beijing, China). Gene-specific primers for qRT-PCR were designed using Primer 3.0 software (https://bioinfo.ut.ee/primer3-0.4.0/, accessed on 15 December, 2022). Information for all the primers used in this study is shown in Table 3. Amplification was conducted on a qTOWER2.2 Real-Time PCR System (Analytik Jena, Jena, Germany). Each qRT-PCR reaction solution contained 1 μL cDNA template, 0.5 μL each forward and reversed primer (200 nM), 5 μL SYBR Green PCR Master Mix (TaKaRa, Dalian, China), and 3 μL nuclease-free water. The qPCR reactions were conducted at 95 °C for 3 min, followed by 40 cycles of 95 °C for 5 s and 60 °C for 30 s. By using *Actin* as an internal reference gene, the relative expression levels of DEGs were calculated using the 2^−ΔΔCT^ method [44]. For qRT-PCR analysis, three biological replications were made for each group.

## 5. Conclusions

**Figure 8:**
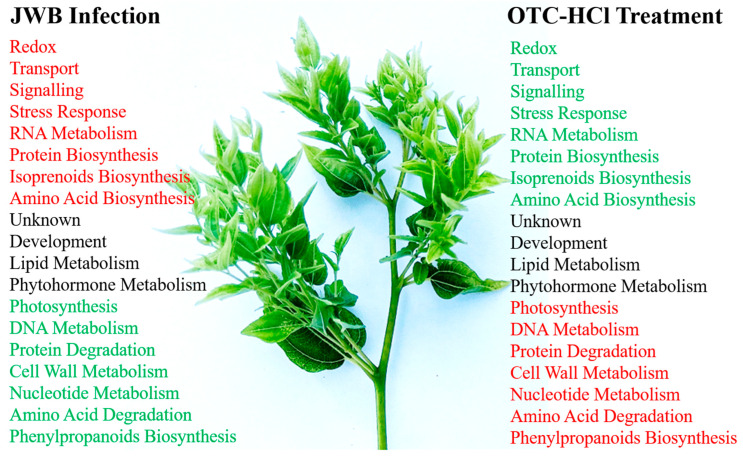
Summary diagram for classifying the chemotherapy effects of OTC-HCl on JWB-diseased jujube trees. JWB infection up-regulated genes were mostly involved in redox, transport, signaling, stress response, RNA metabolism, protein biosynthesis, isoprenoids biosynthesis and amino acid biosynthesis. JWB infection down-regulated genes were mainly involved in photosynthesis, DNA metabolism, protein degradation, cell wall metabolism, nucleotide metabolism, amino acid degradation and phenylpropanoids biosynthesis. JWB phytoplasma infection also greatly influenced the expression of many unknown genes, development, lipid metabolism and phytohormone metabolism-related genes. After OTC-HCl treatment, most of the JWB-diseased trees exhibited healthy morphology, and the expression of most phytoplasma infection-responsive genes recovered to normal levels.

In this study, we reported the chemotherapy effects of OTC-HCl in recovering JWB-diseased trees. Through comparative transcriptomic analysis, we identified 755 DEGs among healthy, JWB-diseased, and OTC-HCl-treated JWB-diseased jujube leaves. Enrichment analysis of DEGs revealed that phytoplasma infection greatly affected the DNA and RNA metabolisms and signaling in jujube leaves. The abnormal changes in the genes involved in photosynthesis, metabolisms of both primary and secondary metabolites and their transportation, and many other pathways might be closely related to the nutrient deficiency symptoms in JWB-diseased jujube leaves. Moreover, the expression of phytohormone metabolism-, lipid metabolism- and development-related genes in jujube leaves was also greatly influenced by phytoplasma infection. Many functional unknown genes and some other pathways-related genes (such as *PAP17*, *NDF1*, *Major allergen Mal d1*, *Mediator complex subunit Med2*, *PPOs*, *GSTs*, *D-arabinono-1,4-lactone oxidase family proteins*, *BURP domain-containing proteins*, etc.) showed significant differential expression in response to both JWB phytoplasma infection and OTC-HCl treatment, whose roles in the jujube–JWB interactions need to be further studied. The abnormal changes in these DEGs mostly recovered after OTC-HCl treatment (Figure 8). The DEGs identified in this study have great potential to be used as valuable gene resources in the jujube JWB resistance breeding in the future.

## Figures and Tables

**Table 1:** Genome alignment results of RNA-Seq data of healthy control (C group), JWB-diseased (D group) and OTC-HCl treated JWB-diseased (T group) *Z. jujuba* cv. ‘Muzao’ leaves. C1-C3, D1-D3 and T1-T3 were three biological replications for C, D and T groups, respectively.

Sample	Total Reads	Mapped Reads	Unique Mapped Reads	Multiple Mapped Reads	Clean Bases	GC (%)	Q20 (%)	Q30 (%)
C1	40,246,416	34,584,165 (85.93%)	30,208,421 (75.06%)	4,375,744 (10.87%)	6,004,362,814	45.49	97.18	92.48
C2	41,676,608	36,205,312 (86.87%)	31,635,868 (75.91%)	4,569,444 (10.96%)	6,232,310,268	45.09	97.24	92.52
C3	42,367,776	36,592,177 (86.37%)	32,020,841 (75.58%)	4,571,336 (10.79%)	6,342,330,282	44.5	97.35	92.66
D1	43,118,146	37,795,114 (87.65%)	33,077,612 (76.71%)	4,717,502 (10.94%)	6,452,953,630	44.56	97.44	92.85
D2	43,524,986	38,115,993 (87.57%)	33,306,163 (76.52%)	4,809,830 (11.05%)	6,515,843,862	44.74	97.54	93.14
D3	43,240,166	37,899,736 (87.65%)	33,175,601 (76.72%)	4,724,135 (10.93%)	6,473,987,776	44.97	97.36	92.76
T1	39,165,042	33,404,398 (85.29%)	28,961,978 (73.95%)	4,442,420 (11.34%)	5,828,462,420	45.33	97.12	92.15
T2	41,538,142	35,253,269 (84.87%)	30,817,826 (74.19%)	4,435,443 (10.68%)	6,192,986,034	45.46	96.93	92.01
T3	42,794,538	37,476,631 (87.57%)	32,751,477 (76.53%)	4,725,154 (11.04%)	6,409,198,938	44.77	97.12	92.19

**Table 2:** MapMan annotation results of the DEGs identified in this study. For proteins with functions in diverse biological processes, multiple Bins were assigned. The number in bold represents that this pathway is significantly enriched.

Bin	Bin Description	Elements	*p*-Value in C vs. D	*p*-Value in C vs. T	*p*-Value in D vs. T
35	not assigned	196	7.21 × 10^−1^	8.79 × 10^−1^	9.89 × 10^−1^
26	misc	88	7.21 × 10^−1^	9.72 × 10^−1^	7.70 × 10^−1^
27	RNA	72	9.93 × 10^−1^	8.52 × 10^−1^	7.25 × 10^−1^
30	signaling	65	**2.34 × 10^−3^**	8.43 × 10^−1^	**6.83 × 10^−3^**
29	protein	62	9.72 × 10^−1^	6.57 × 10^−1^	7.58 × 10^−1^
20	stress	49	8.47 × 10^−1^	8.52 × 10^−1^	9.46 × 10^−1^
34	transport	42	**4.93 × 10^−3^**	6.02 × 10^−1^	**2.33 × 10^−4^**
16	secondary metabolism	37	8.44 × 10^−1^	8.33 × 10^−1^	9.46 × 10^−1^
17	hormone metabolism	37	7.86 × 10^−1^	4.83 × 10^−1^	7.54 × 10^−1^
28	DNA	22	**2.34 × 10^−3^**	9.72 × 10^−1^	**4.65 × 10^−2^**
10	cell wall	20	3.07 × 10^−1^	8.10 × 10^−1^	6.45 × 10^−1^
11	lipid metabolism	20	8.43 × 10^−1^	9.26 × 10^−1^	8.63 × 10^−1^
31	cell	19	7.21 × 10^−1^	9.75 × 10^−1^	7.70 × 10^−1^
1	PS	17	3.51 × 10^−1^	1.94 × 10^−1^	**1.01× 10^−2^**
33	development	16	7.33 × 10^−1^	6.57 × 10^−1^	9.46 × 10^−1^
13	amino acid metabolism	9	8.46 × 10^−1^	7.04 × 10^−1^	8.63 × 10^−1^
21	redox	9	7.21 × 10^−1^	9.88 × 10^−1^	7.43 × 10^−1^
23	nucleotide metabolism	7	7.21 × 10^−1^	8.13 × 10^−1^	5.89 × 10^−1^
18	Cofactor and vitamin metabolism	5	7.21 × 10^−1^	7.14 × 10^−1^	5.64 × 10^−1^
2	major CHO metabolism	3	7.21 × 10^−1^	9.75 × 10^−1^	8.90 × 10^−1^
4	glycolysis	3	7.36 × 10^−1^	7.04 × 10^−1^	9.63 × 10^−1^
9	mitochondrial electron transport/ATP synthesis	3	1.00	9.34 × 10^−1^	9.34 × 10^−1^
7	OPP	2	6.68 × 10^−1^	9.72 × 10^−1^	6.45 × 10^−1^
15	metal handling	2	9.44 × 10^−1^	9.76 × 10^−1^	9.89 × 10^−1^
24	Biodegradation of Xenobiotics	2	7.36 × 10^−1^	8.52 × 10^−1^	8.90 × 10^−1^
3	minor CHO metabolism	1	7.21 × 10^−1^	8.36 × 10^−1^	6.45 × 10^−1^
14	S-assimilation	1	8.01 × 10^−1^	9.26 × 10^−1^	8.19 × 10^−1^
22	polyamine metabolism	1	9.82 × 10^−1^	8.58 × 10^−1^	9.18 × 10^−1^
25	C1-metabolism	1	7.21 × 10^−1^	6.93 × 10^−1^	9.89 × 10^−1^

**Table 3:** Information for the primers used in this study. CNGC: cyclic nucleotide-gated channel; ABC2: ATP-binding cassette transporter 2; GAD: Glutamate decarboxylase; CaBP: calcium-binding EF-hand family protein; TC: terpenoid cyclase; TS: terpene synthase (TS); DRP: CC-NBS-LRR class disease resistance protein.

Gene Name	Gene ID	Forward Primer (5′→3′)	Reverse Primer (5′→3′)
*Actin*	*-*	CTTGCATCCCTCAGCACCTT	TCCTGTGGACAATGGATGGA
*CNGC*	gene7282	GATGCTTTTCATCACCCA	TGTGGTTTTGGAACCATC
*ABC2*	gene9336	TGTGCAAGAGTTGAGGAA	TTCGCTGGACTCTGTATG
*GAD*	gene11898	GGTAGAGCTGAAAGAGGTA	GGCTGCTACACATATAGTG
*CaBP*	gene13237	GAGGAGCTTAACTGGCTA	CATGGTTTTCCCACAGAG
*TC*	gene14697	ACTGCAATAGGATTGATGG	GCTCGATGTATAGGAGTTG
*TS*	gene14606	CAGTCGGTATATTACTATTGGA	GCTTCTTAAAATATTCACCATTG
*DRP*	gene25582	GAGGGTTTCCAAGTCTCA	GCCTTTCGAGATGAGGTA
*Unknown*	gene19644	CTTGGTTTGGATGACTTTG	CCTCTTTCAAAACACTCAC

## Data Availability

All data are available in this article and in the supplementary files.

## Supplementary Materials

The supporting information can be downloaded at: https://www.mdpi.com/article/10.3390/ijms241210313/s1.
