# Supplementary figures and images for: Influences of Jujube Witches’ Broom (JWB) Phytoplasma Infection and Oxytetracycline Hydrochloride Treatment on the Gene Expression Profiling in Jujube
Source: Int J Mol Sci. 2023 Jun 18;24(12):10313. doi: 10.3390/ijms241210313 (PMC10299734; doi:10.3390/ijms241210313)

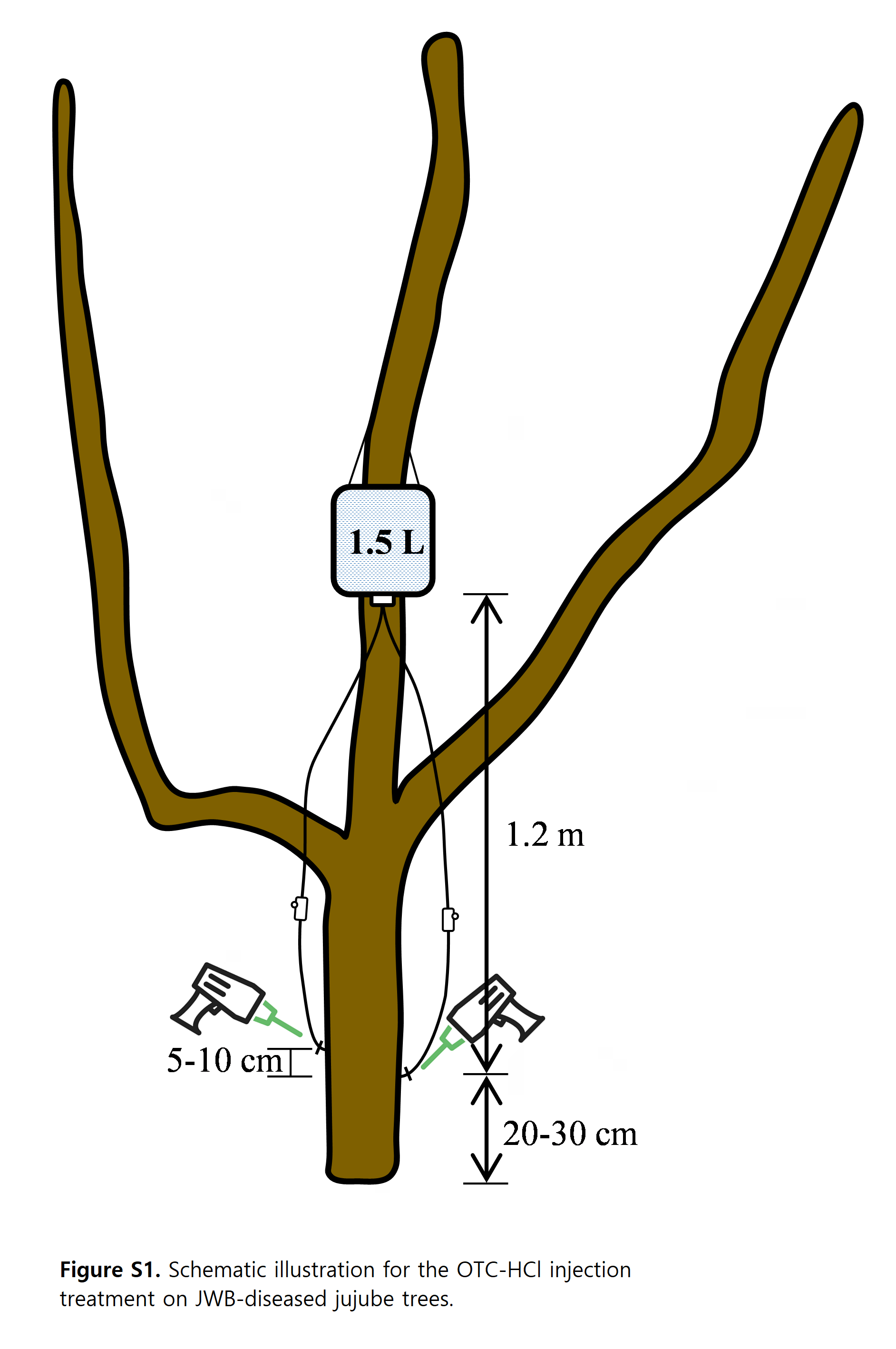

Supplement: Supplementary file 1 [file ijms-24-10313-s001.zip › Supplemental Figure S1.png]
